# Supplementary figures and images for: The Arp2/3 complex is required for in situ haptotactic response of microglia to iC3b
Source: EMBO Rep. 2026 Feb 27;27(7):1666–95. doi: 10.1038/s44319-026-00720-9 (PMC13076747; doi:10.1038/s44319-026-00720-9)

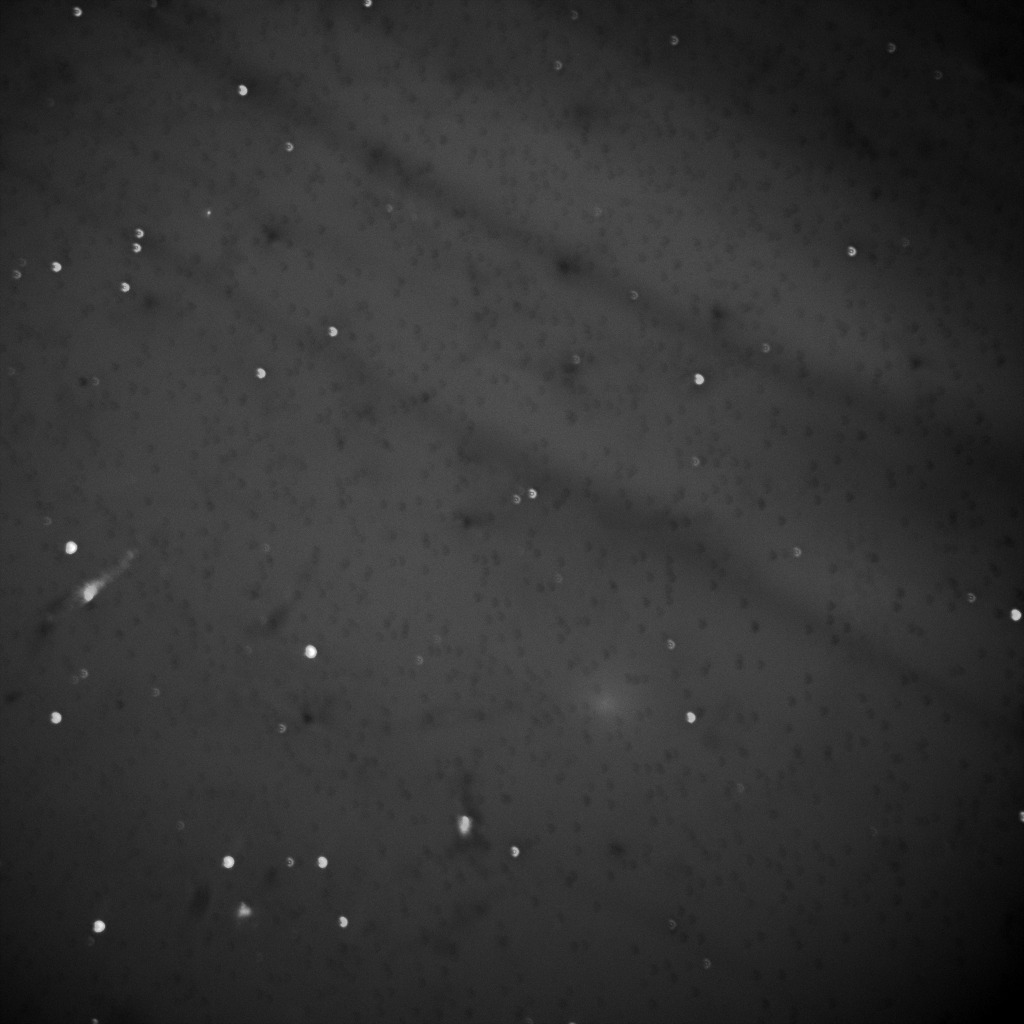

Supplement: Supplementary file 13 — Source data Fig. 1 [file 44319_2026_720_MOESM13_ESM.zip › Figure 1/1A-E/1 ug per mL iC3b 0h.png]

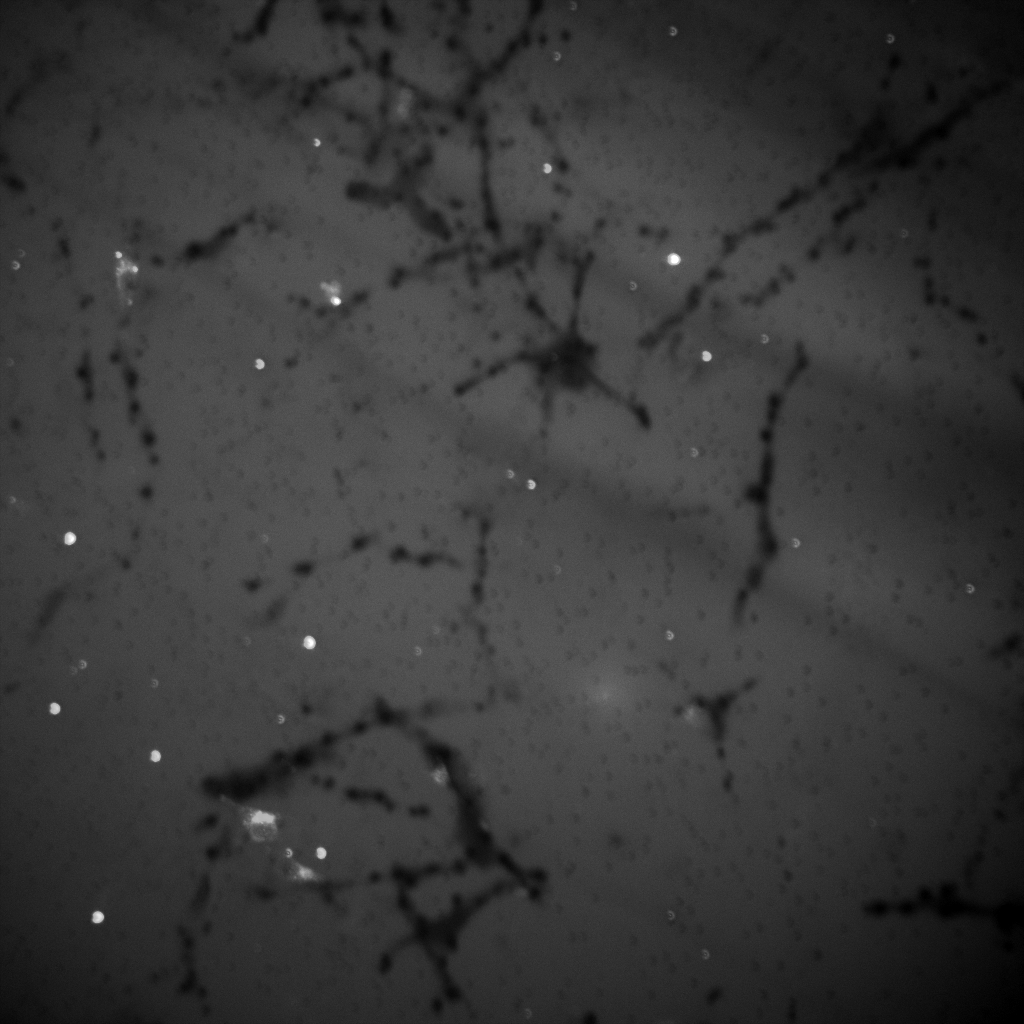

Supplement: Supplementary file 13 — Source data Fig. 1 [file 44319_2026_720_MOESM13_ESM.zip › Figure 1/1A-E/1 ug per mL iC3b 16h.png]

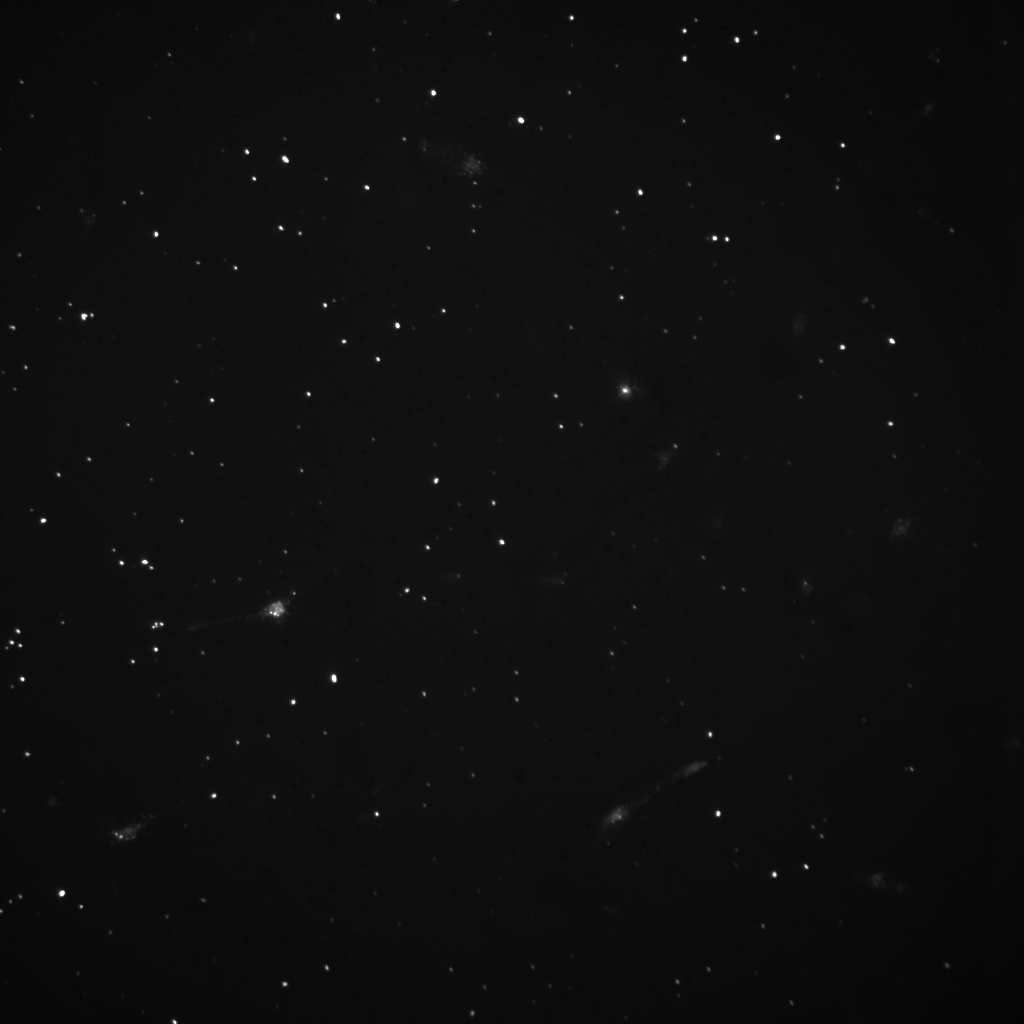

Supplement: Supplementary file 13 — Source data Fig. 1 [file 44319_2026_720_MOESM13_ESM.zip › Figure 1/1A-E/10 ug per mL iC3b 0h.png]

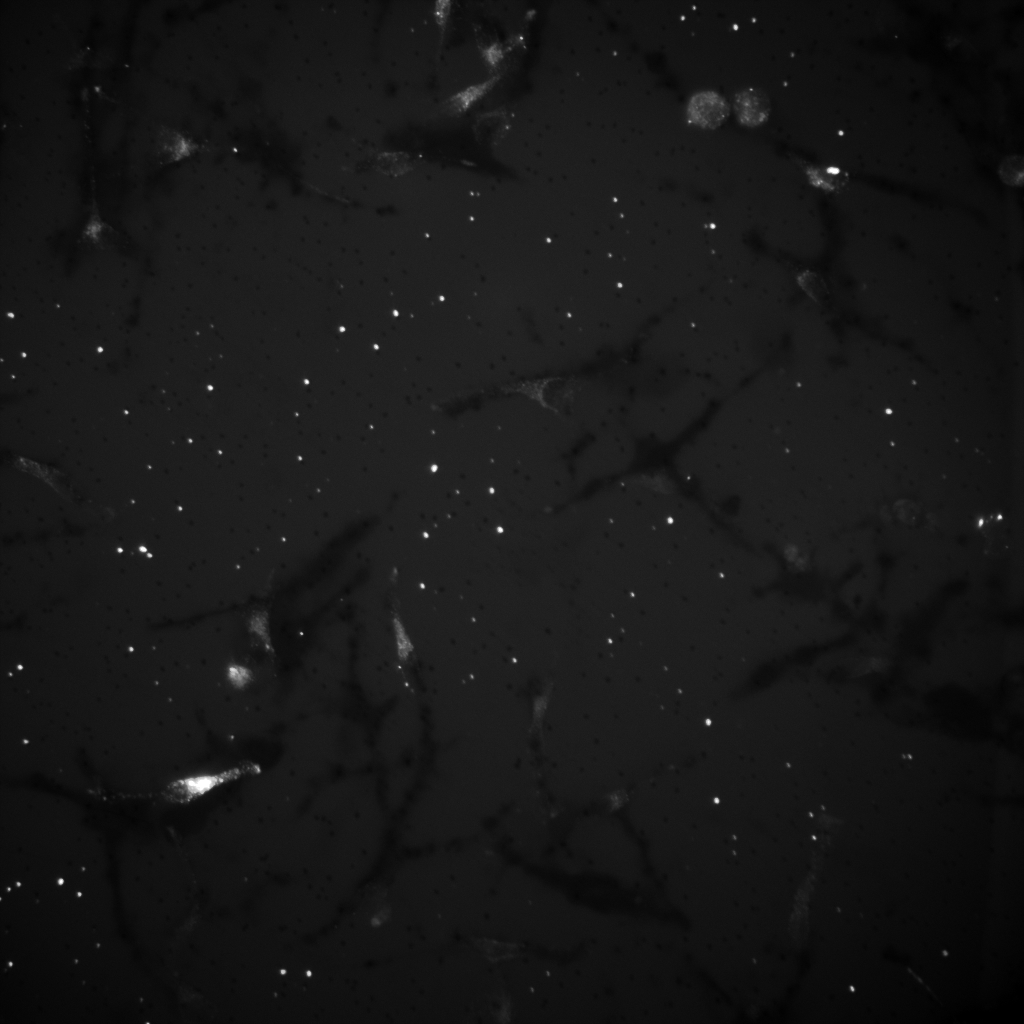

Supplement: Supplementary file 13 — Source data Fig. 1 [file 44319_2026_720_MOESM13_ESM.zip › Figure 1/1A-E/10 ug per mL iC3b 16h.png]

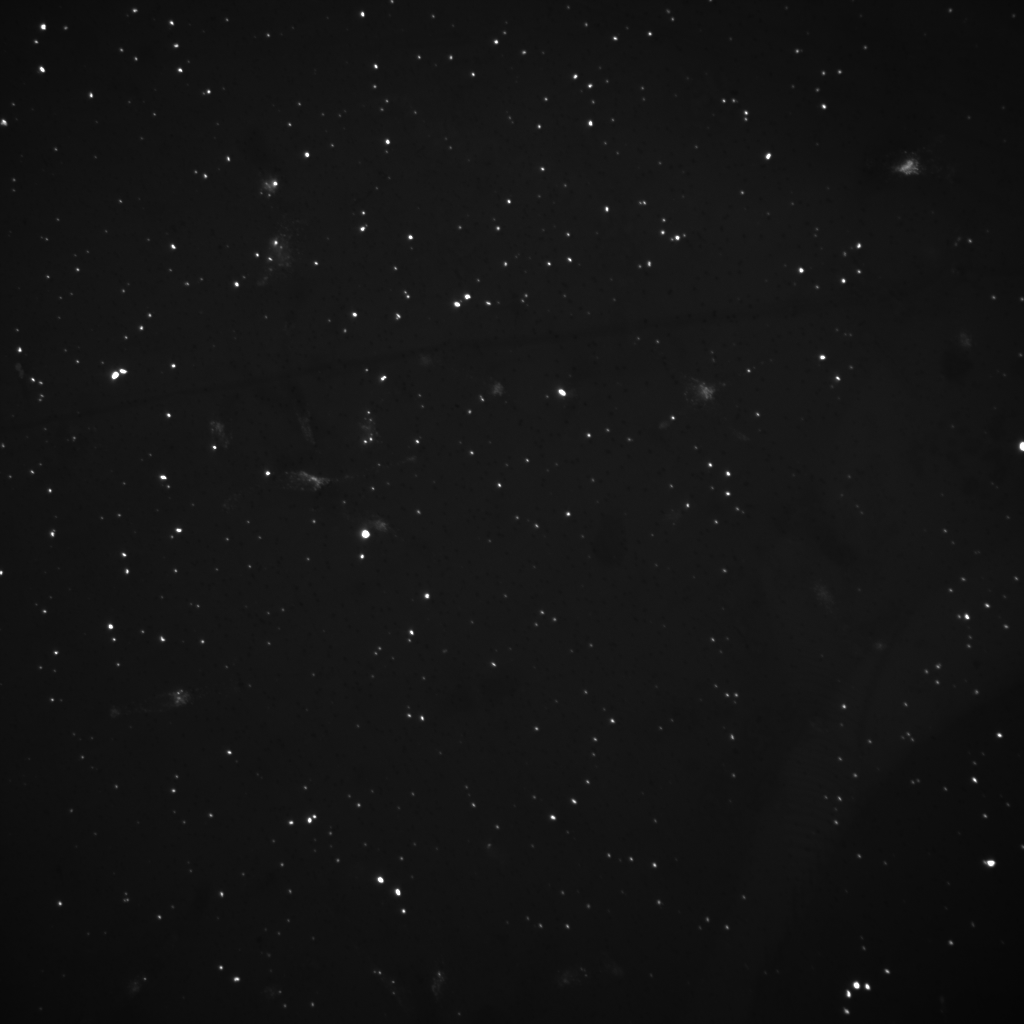

Supplement: Supplementary file 13 — Source data Fig. 1 [file 44319_2026_720_MOESM13_ESM.zip › Figure 1/1A-E/20 ug per mL iC3b 0h.png]

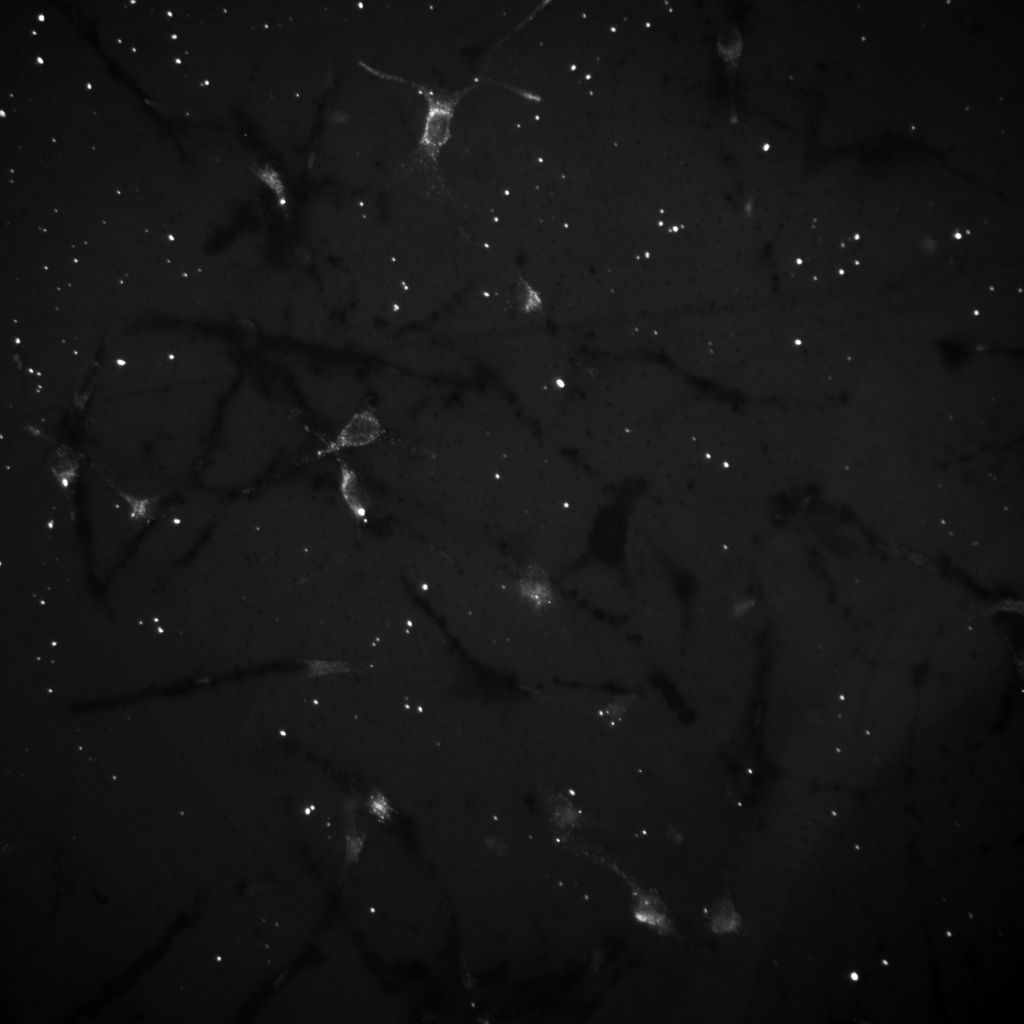

Supplement: Supplementary file 13 — Source data Fig. 1 [file 44319_2026_720_MOESM13_ESM.zip › Figure 1/1A-E/20 ug per mL iC3b 16h.png]

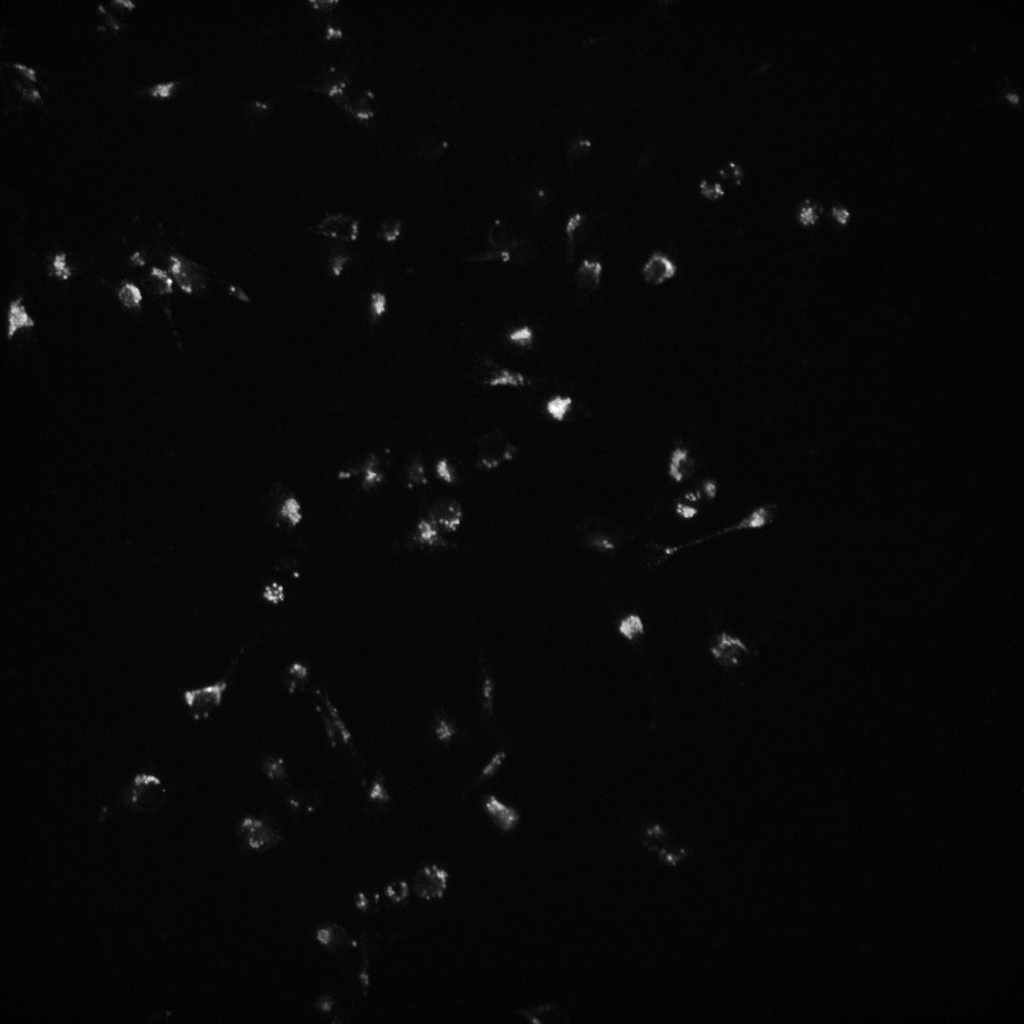

Supplement: Supplementary file 13 — Source data Fig. 1 [file 44319_2026_720_MOESM13_ESM.zip › Figure 1/1F-J/Confined iC3b channel.png]

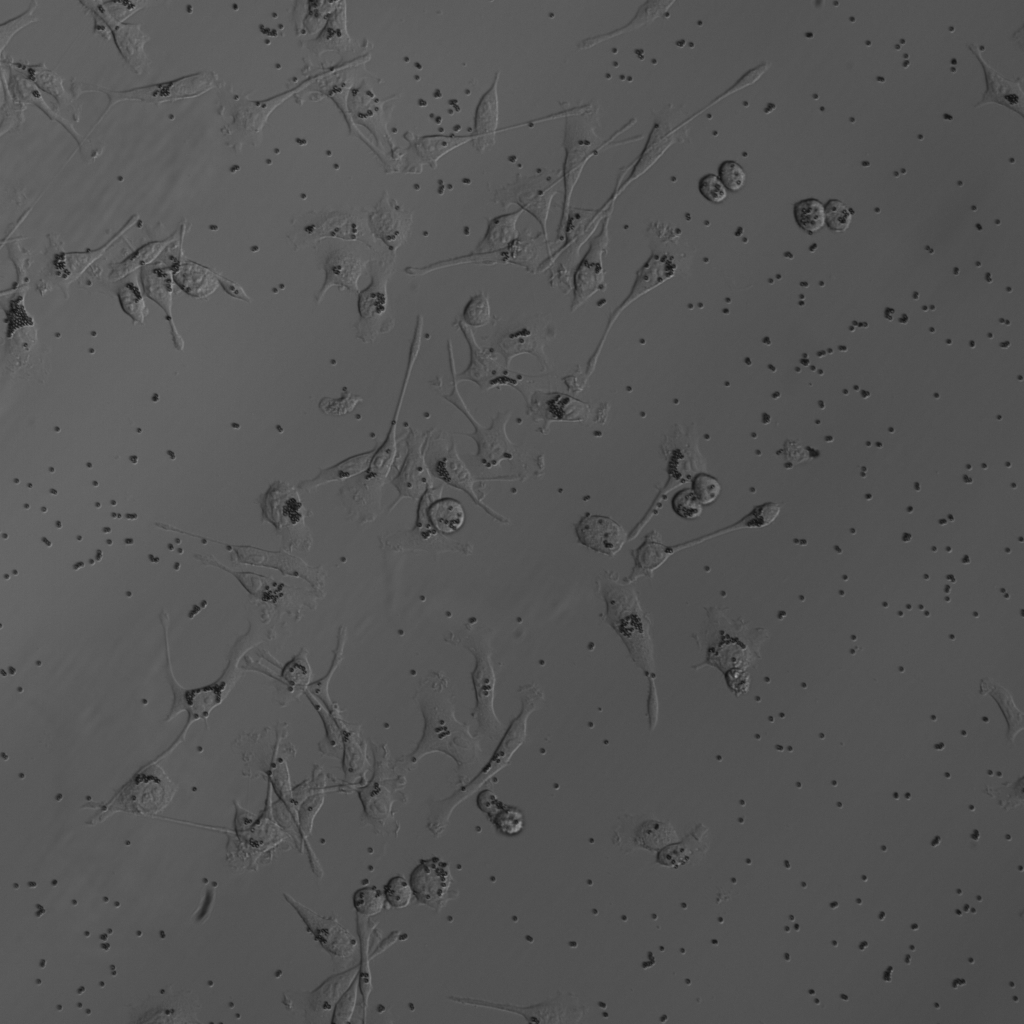

Supplement: Supplementary file 13 — Source data Fig. 1 [file 44319_2026_720_MOESM13_ESM.zip › Figure 1/1F-J/Confined relief contrast.png]

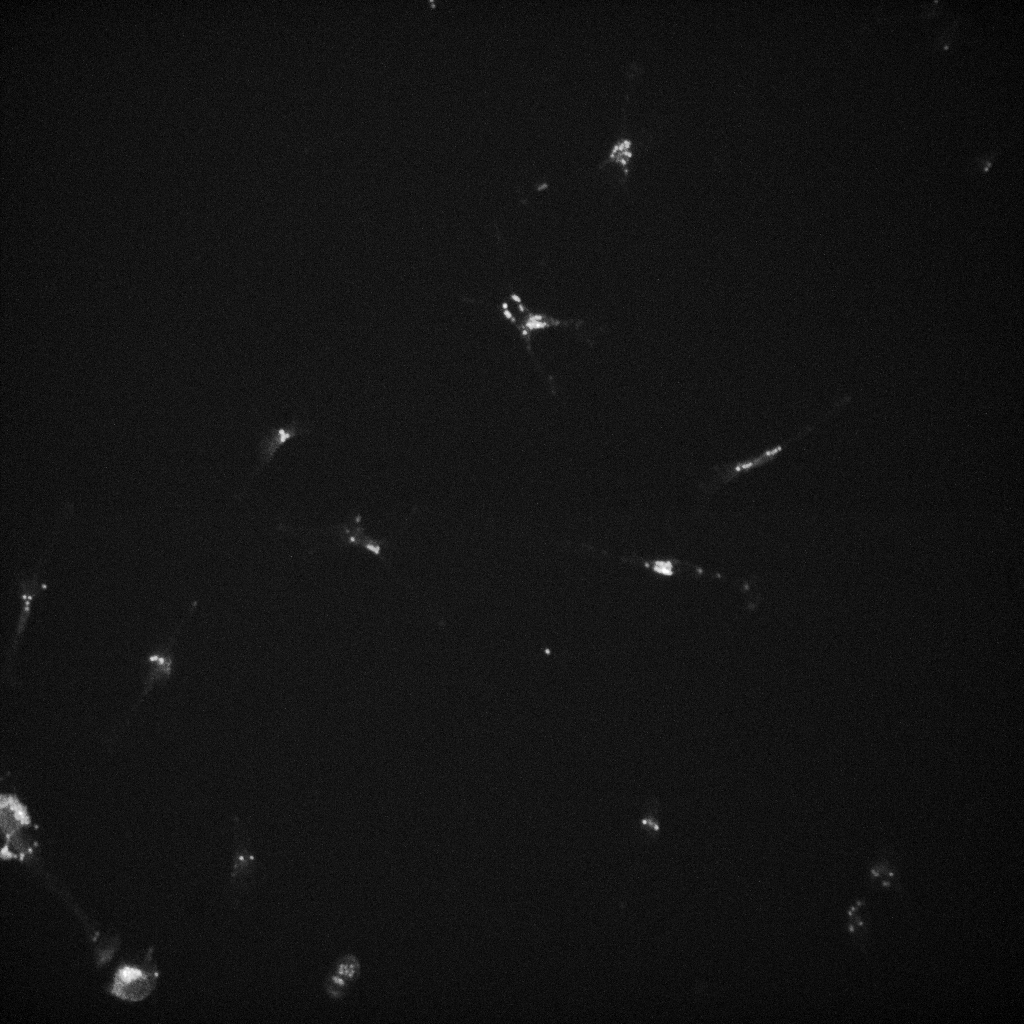

Supplement: Supplementary file 13 — Source data Fig. 1 [file 44319_2026_720_MOESM13_ESM.zip › Figure 1/1F-J/Media iC3b channel.png]

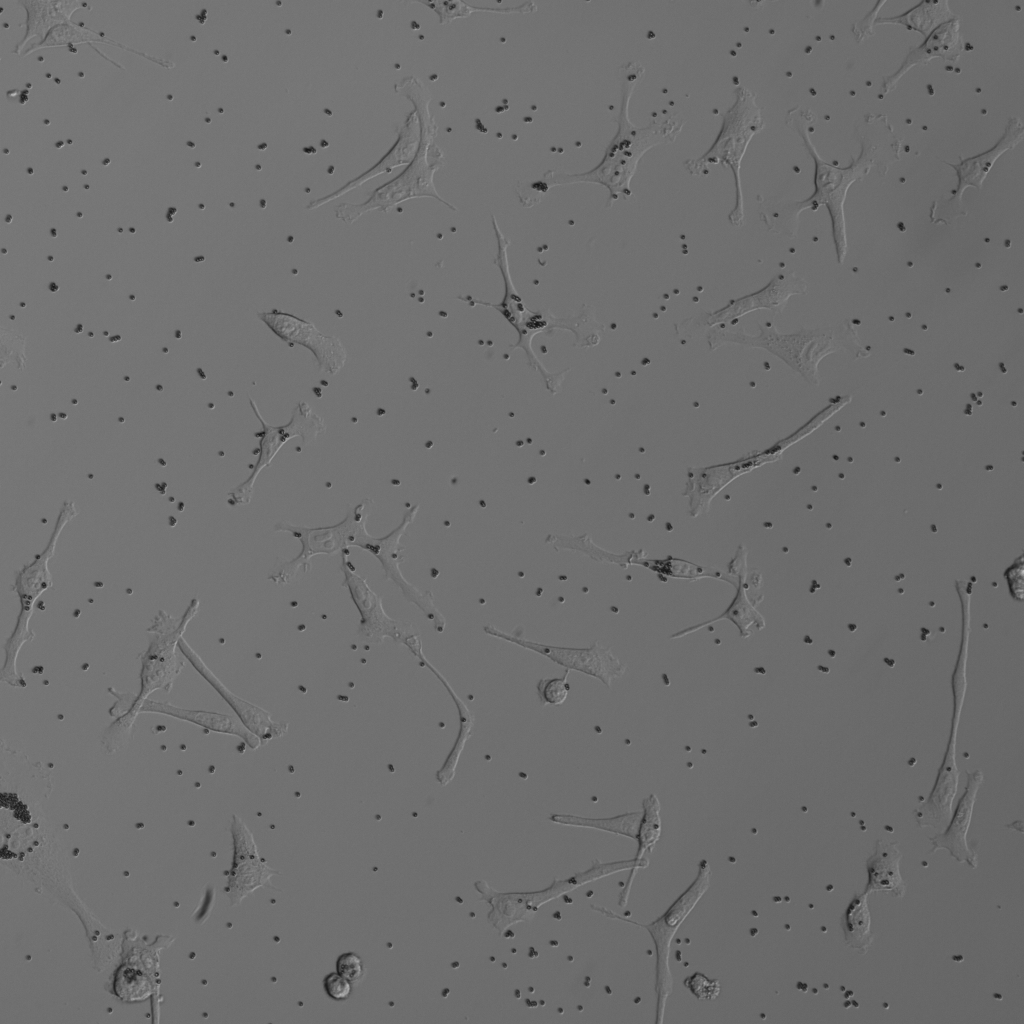

Supplement: Supplementary file 13 — Source data Fig. 1 [file 44319_2026_720_MOESM13_ESM.zip › Figure 1/1F-J/Media Relief Contrast.png]

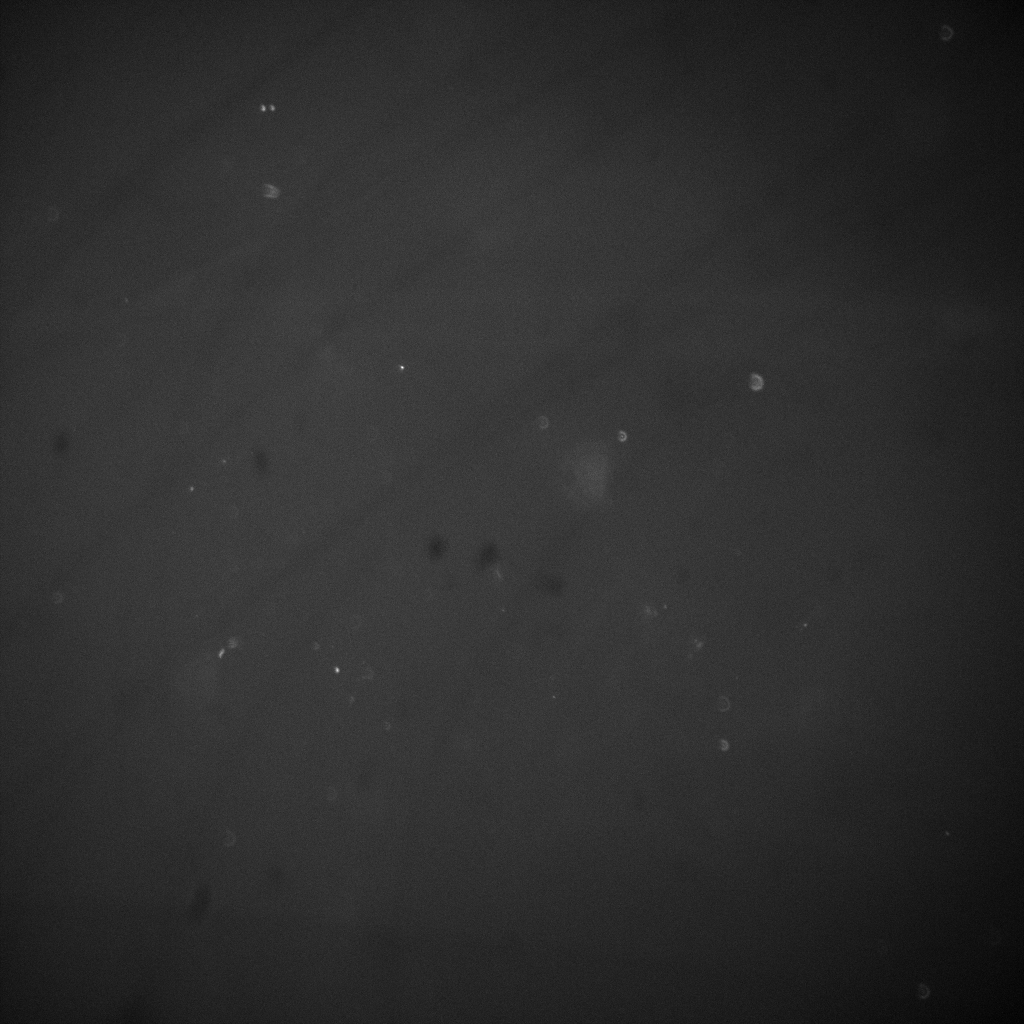

Supplement: Supplementary file 14 — Source data Fig. 2 [file 44319_2026_720_MOESM14_ESM.zip › Figure 2/2E/Confined CK666 0h.png]

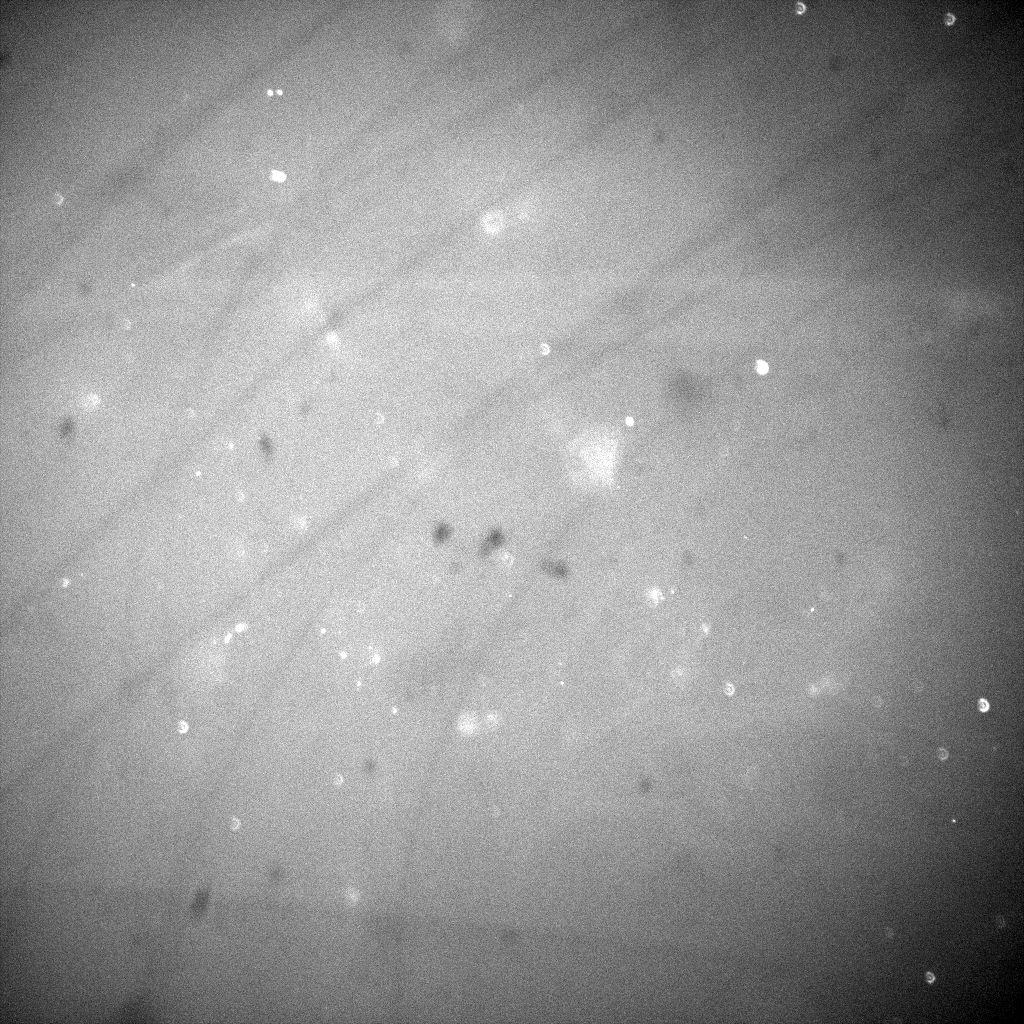

Supplement: Supplementary file 14 — Source data Fig. 2 [file 44319_2026_720_MOESM14_ESM.zip › Figure 2/2E/Confined CK666 16h.png]

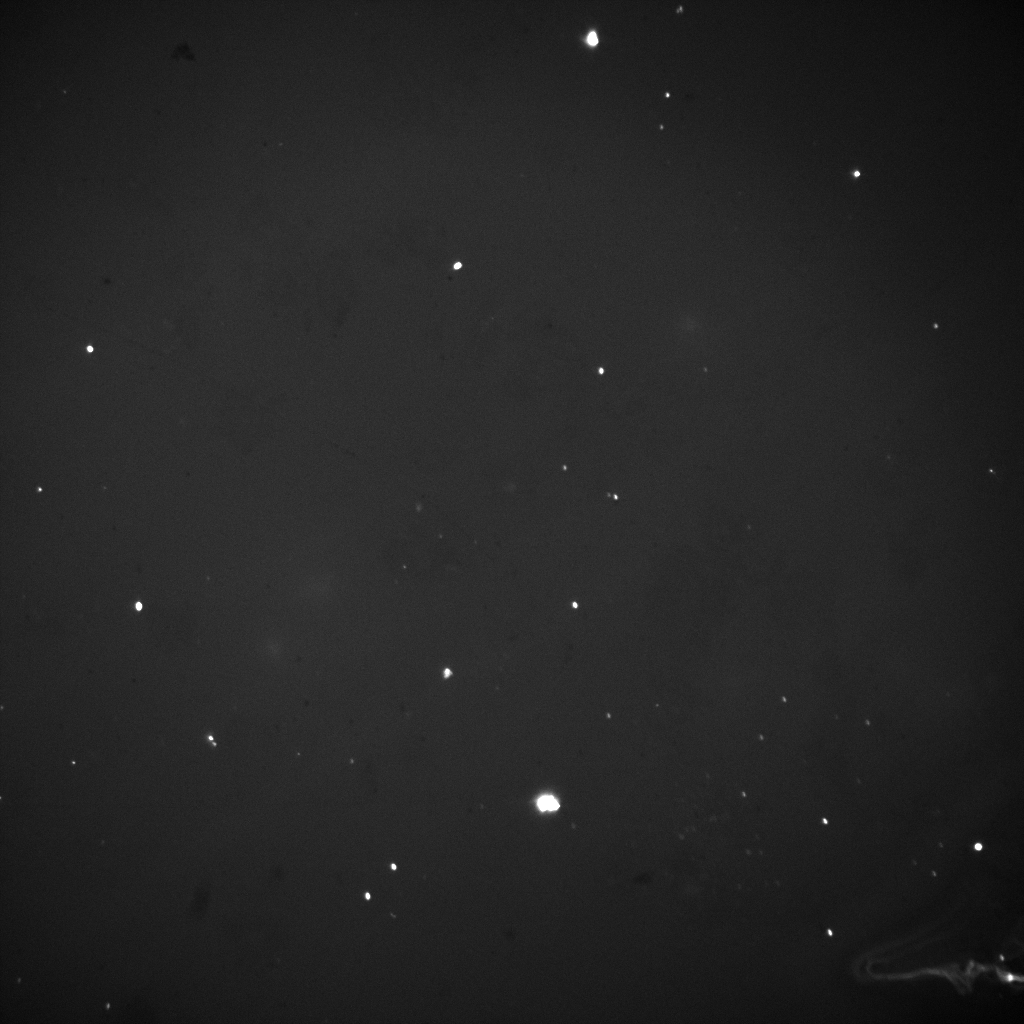

Supplement: Supplementary file 14 — Source data Fig. 2 [file 44319_2026_720_MOESM14_ESM.zip › Figure 2/2E/Confined DMSO 0h.png]

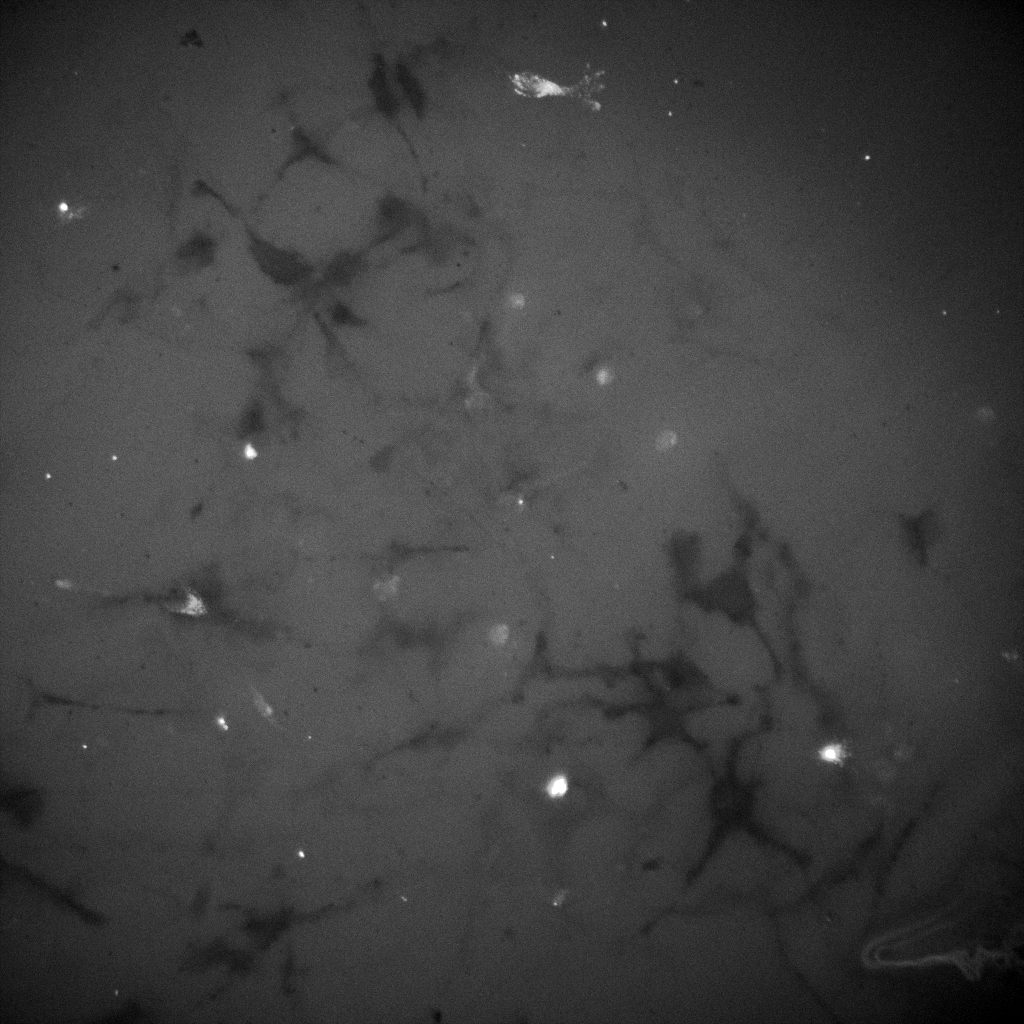

Supplement: Supplementary file 14 — Source data Fig. 2 [file 44319_2026_720_MOESM14_ESM.zip › Figure 2/2E/Confined DMSO 16h.png]

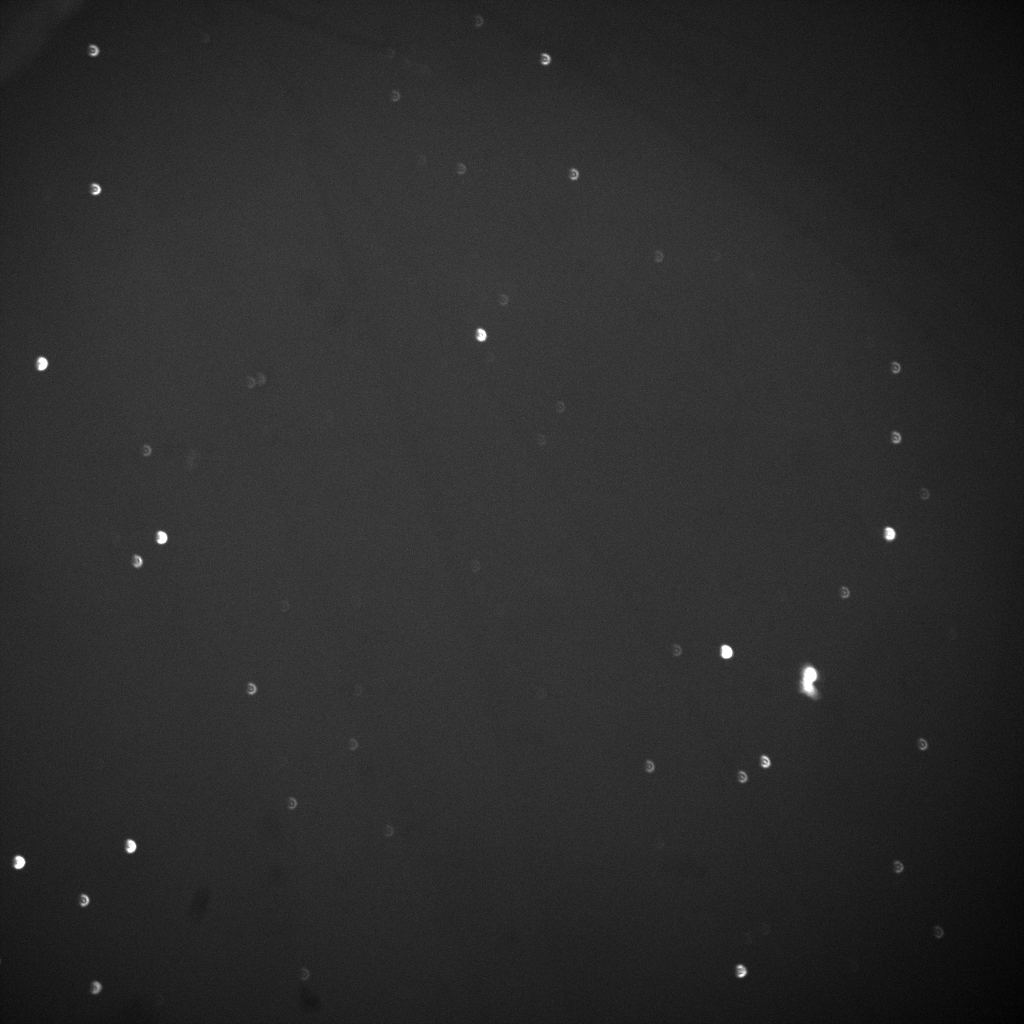

Supplement: Supplementary file 14 — Source data Fig. 2 [file 44319_2026_720_MOESM14_ESM.zip › Figure 2/2E/Media CK666 0h.png]

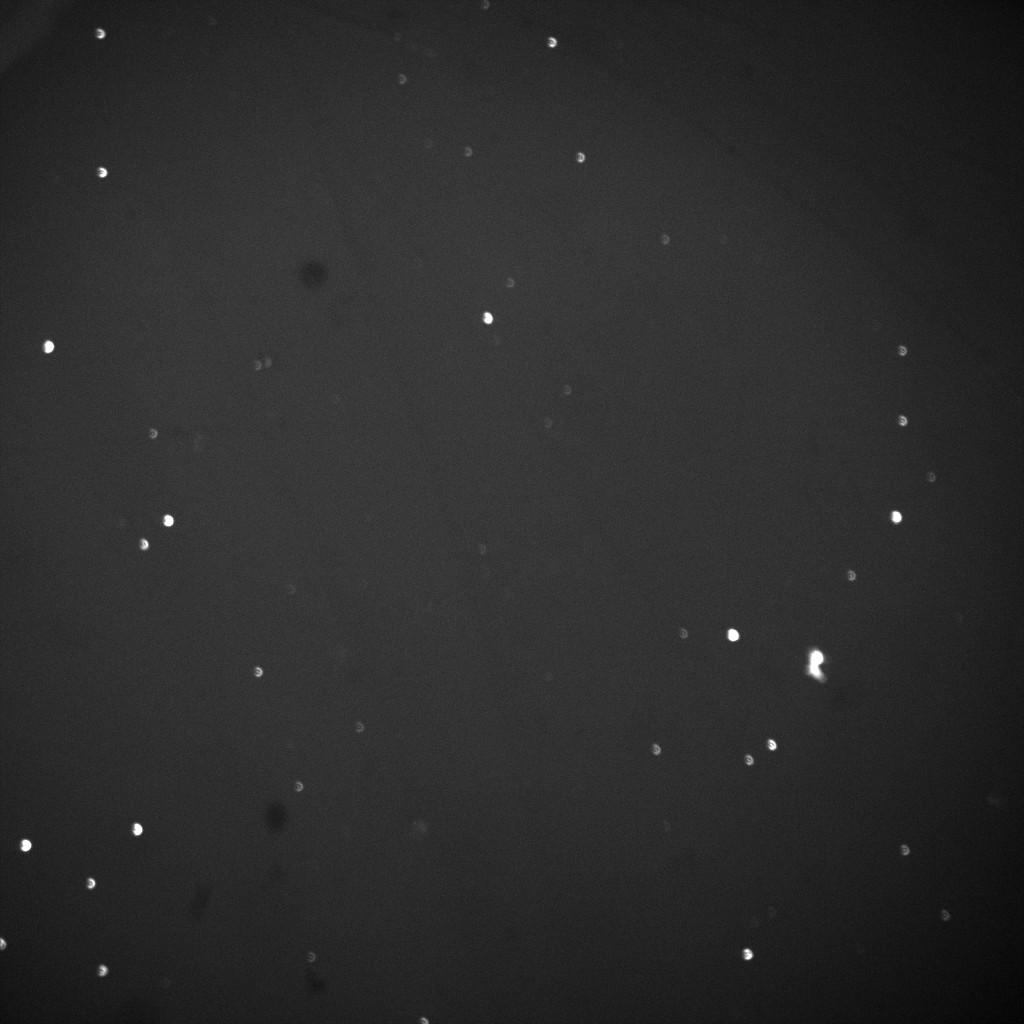

Supplement: Supplementary file 14 — Source data Fig. 2 [file 44319_2026_720_MOESM14_ESM.zip › Figure 2/2E/Media CK666 16h.png]

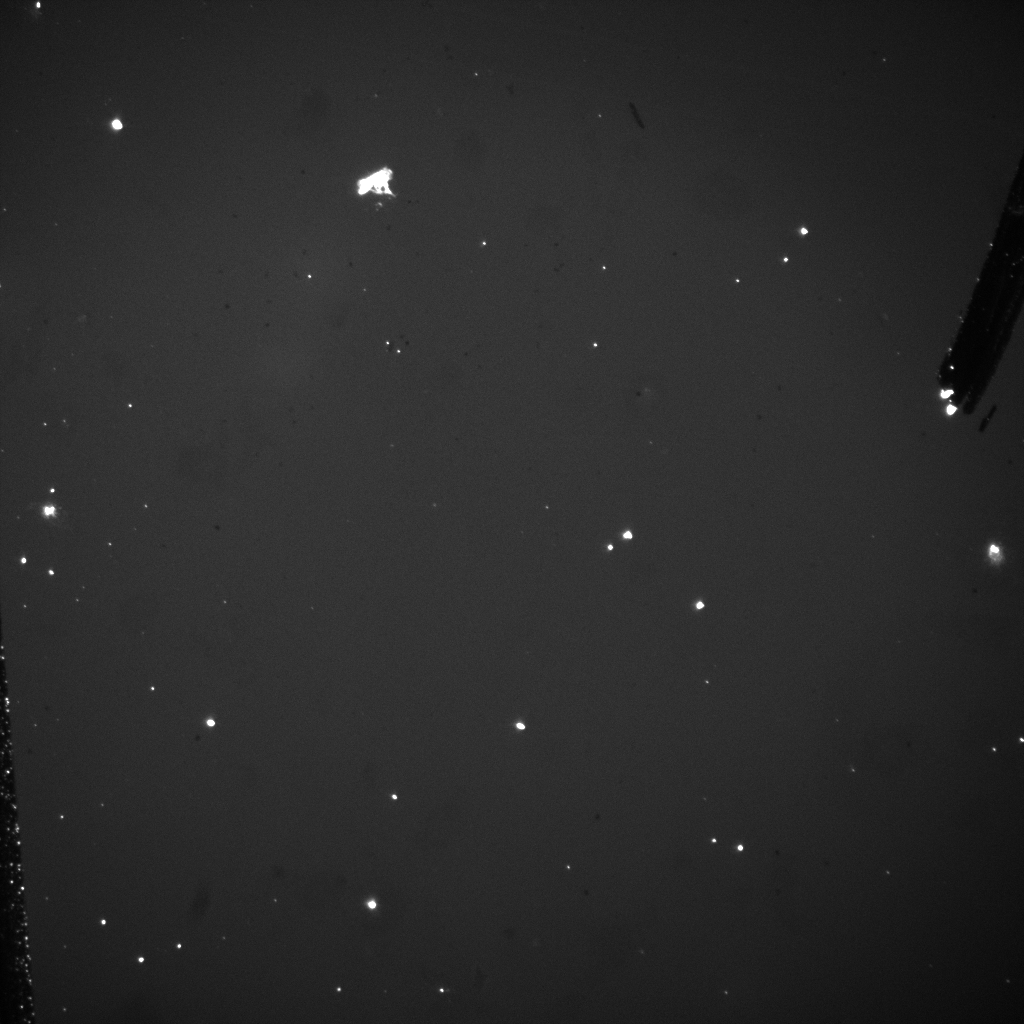

Supplement: Supplementary file 14 — Source data Fig. 2 [file 44319_2026_720_MOESM14_ESM.zip › Figure 2/2E/Media DMSO 0h.png]

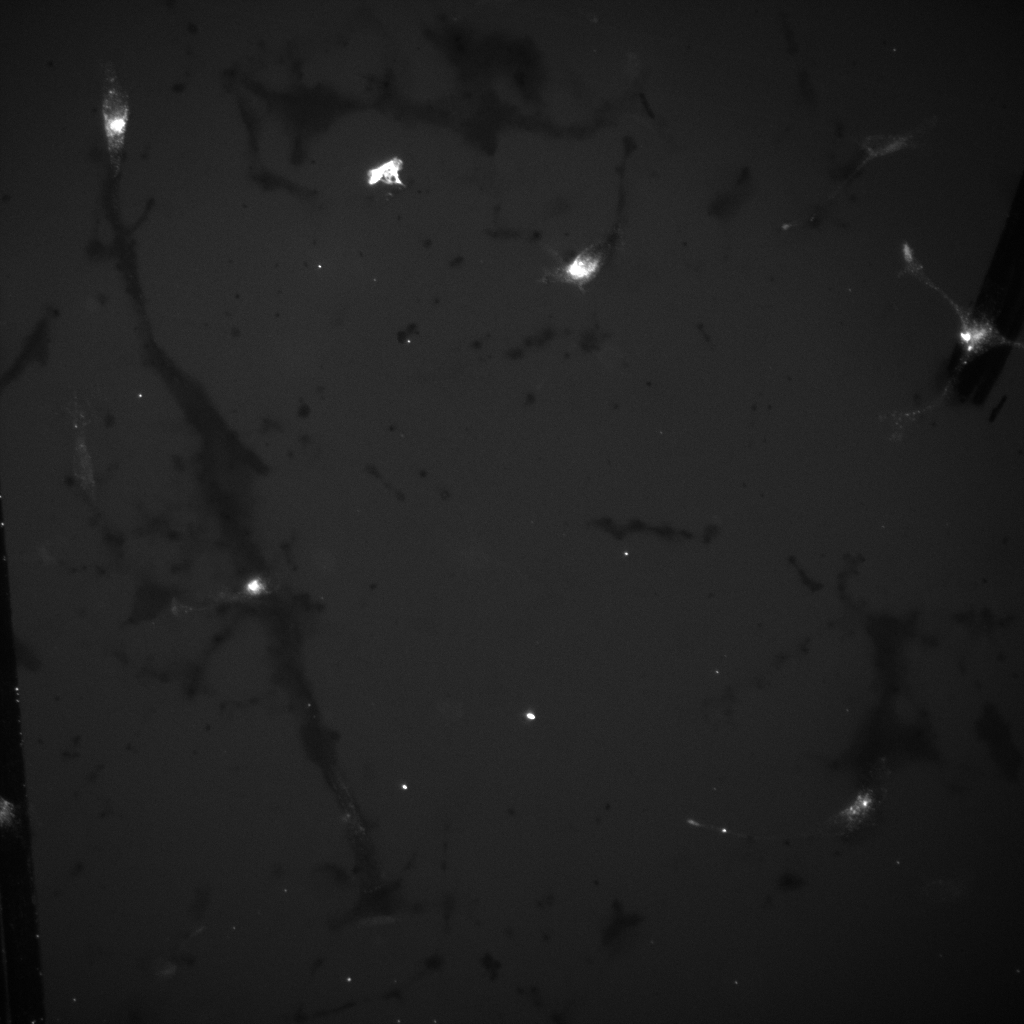

Supplement: Supplementary file 14 — Source data Fig. 2 [file 44319_2026_720_MOESM14_ESM.zip › Figure 2/2E/Media DMSO 16h.png]

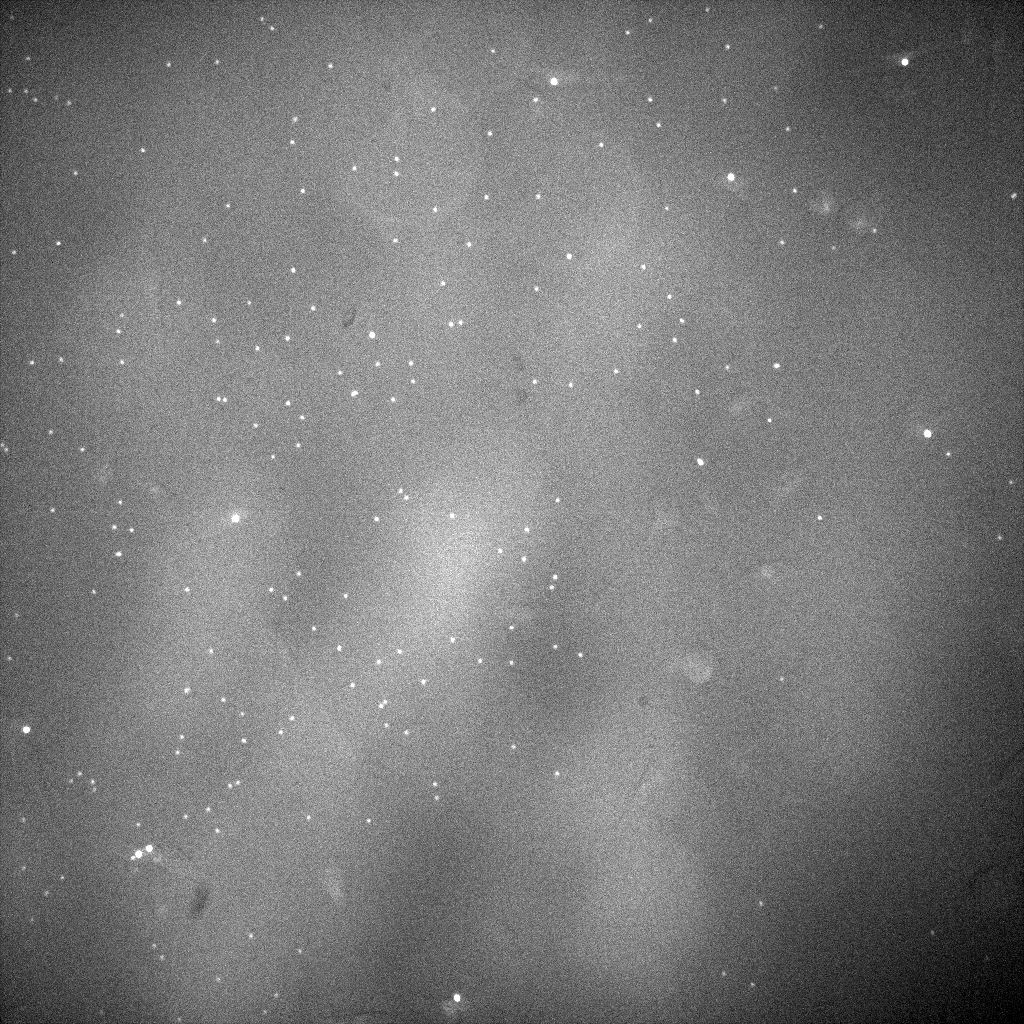

Supplement: Supplementary file 14 — Source data Fig. 2 [file 44319_2026_720_MOESM14_ESM.zip › Figure 2/2F-J/Fig 2J/Confined CK666.png]

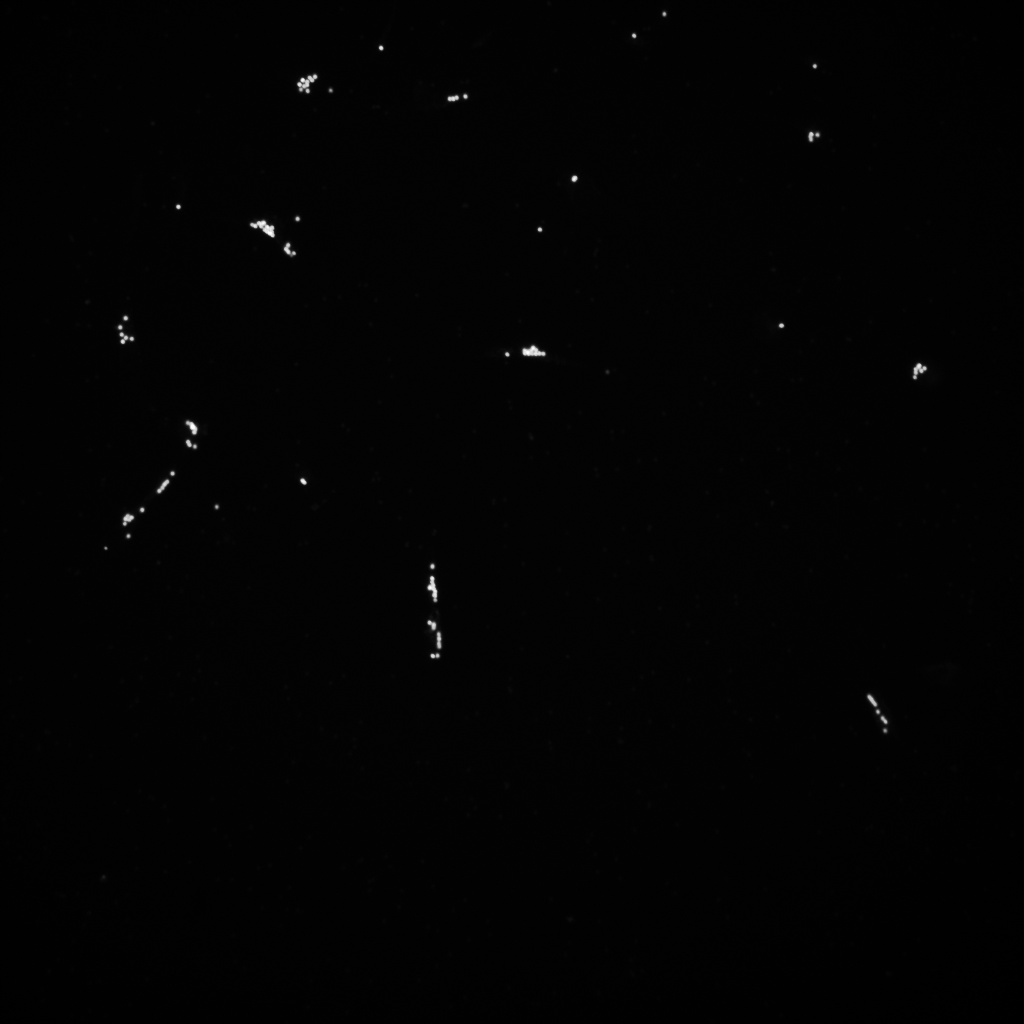

Supplement: Supplementary file 14 — Source data Fig. 2 [file 44319_2026_720_MOESM14_ESM.zip › Figure 2/2F-J/Fig 2J/Confined_DMSO.png]

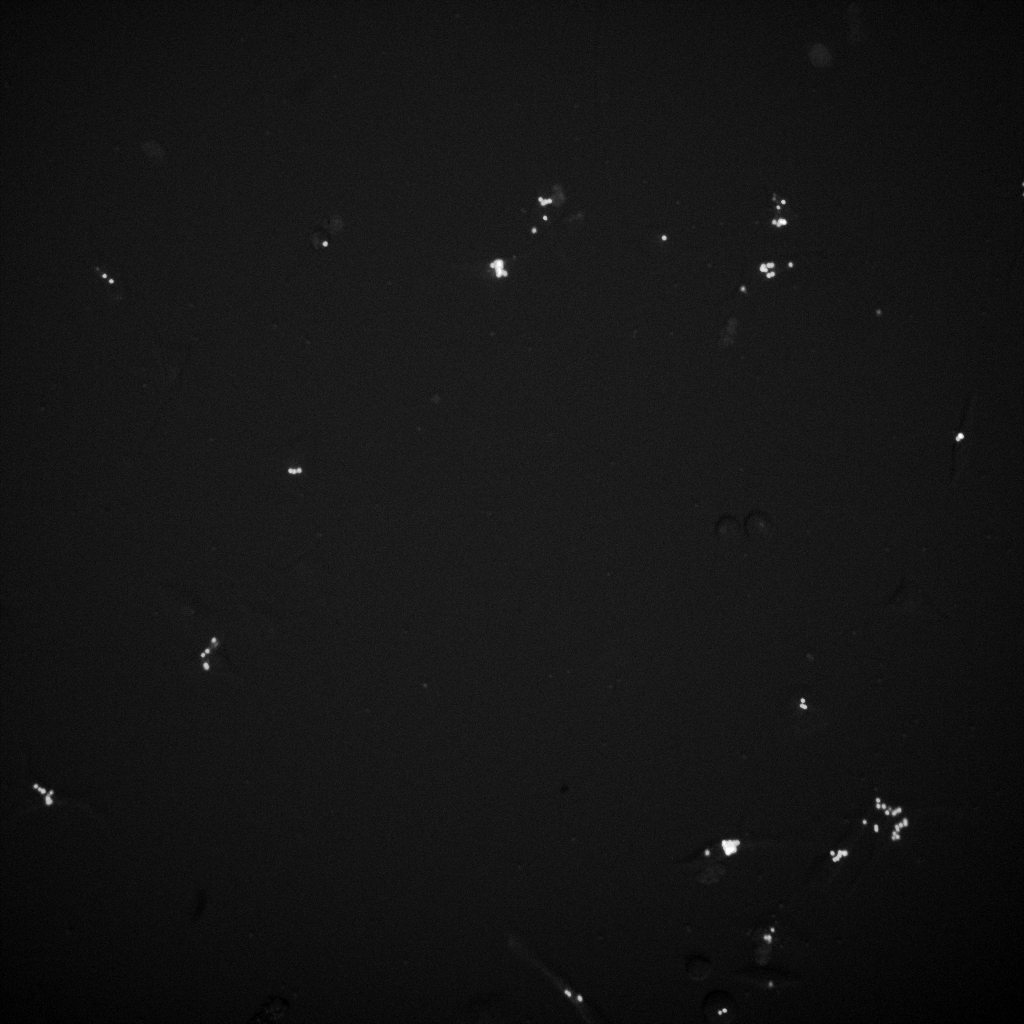

Supplement: Supplementary file 14 — Source data Fig. 2 [file 44319_2026_720_MOESM14_ESM.zip › Figure 2/2F-J/Fig 2J/Media CK666.png]

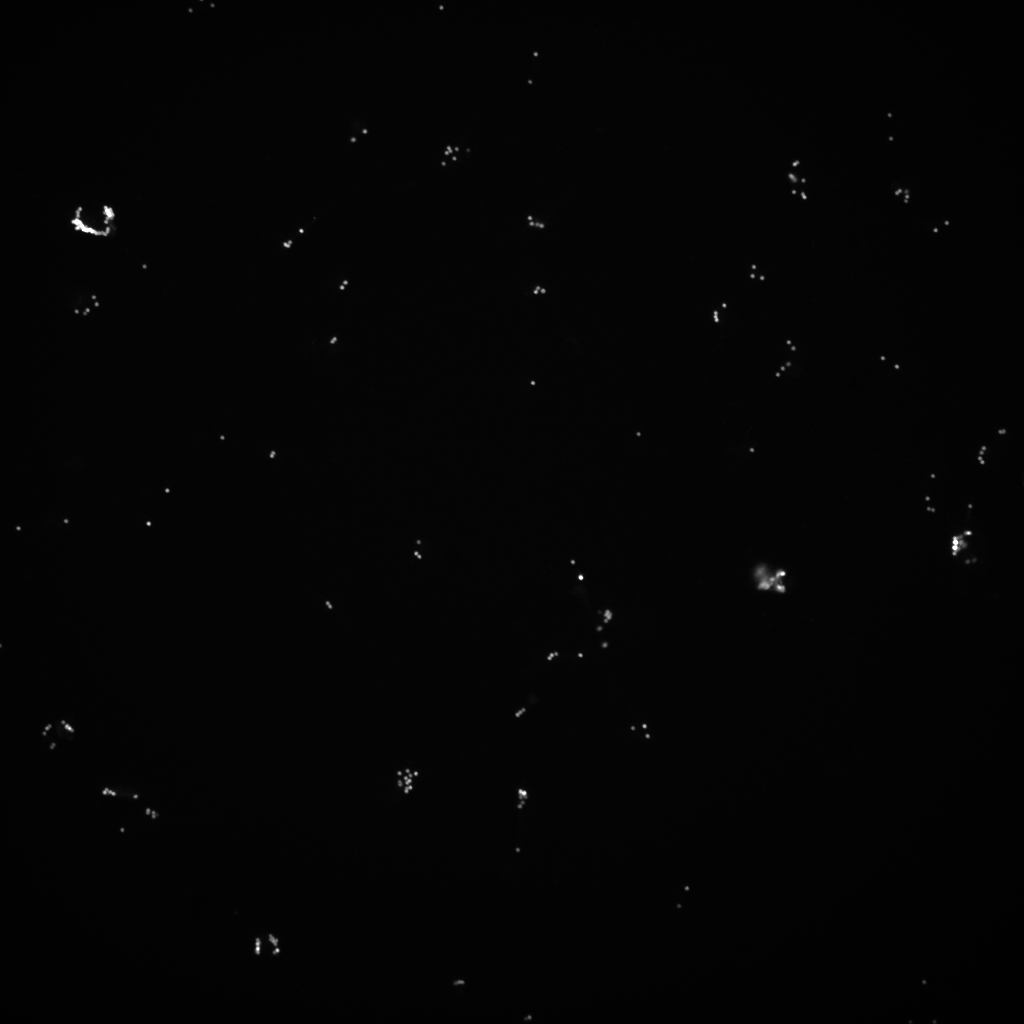

Supplement: Supplementary file 14 — Source data Fig. 2 [file 44319_2026_720_MOESM14_ESM.zip › Figure 2/2F-J/Fig 2J/Media_DMSO.png]

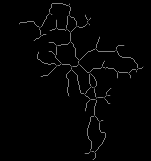

Supplement: Supplementary file 16 — Source data Fig. 4 [file 44319_2026_720_MOESM16_ESM.zip › Figure 4/4A/M2D_Skl_CK.tif]

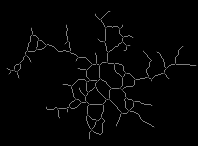

Supplement: Supplementary file 16 — Source data Fig. 4 [file 44319_2026_720_MOESM16_ESM.zip › Figure 4/4A/M2D_Skl_DMSO.tif]

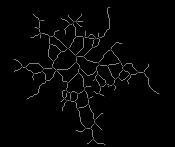

Supplement: Supplementary file 16 — Source data Fig. 4 [file 44319_2026_720_MOESM16_ESM.zip › Figure 4/4A/M2D_Skl_no treat.tif]

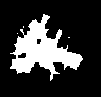

Supplement: Supplementary file 16 — Source data Fig. 4 [file 44319_2026_720_MOESM16_ESM.zip › Figure 4/4E/mean mask_021025 M 200uM CK666 10 min cell 8 9 10_Maximum intensity projection_CUT_250310_120024_X0_Y39_Z0_BIN_250311_205408.tif]

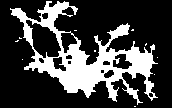

Supplement: Supplementary file 16 — Source data Fig. 4 [file 44319_2026_720_MOESM16_ESM.zip › Figure 4/4E/mean mask_021025 M no treat 10 min cell 2 3_Maximum intensity projection_CUT_250310_101522_X14_Y0_Z0.tif]

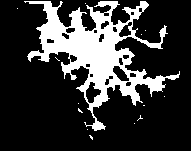

Supplement: Supplementary file 16 — Source data Fig. 4 [file 44319_2026_720_MOESM16_ESM.zip › Figure 4/4E/mean_mask_021025 M DMSO 10 min cell 2 3_Maximum intensity projection_CUT_250311_201820_X76_Y0_z0.tif]
