# Supplementary material for: The Arp2/3 complex is required for in situ haptotactic response of microglia to iC3b
Source: EMBO Rep. 2026 Feb 27;27(7):1666–95. doi: 10.1038/s44319-026-00720-9 (PMC13076747; doi:10.1038/s44319-026-00720-9)
Supplement: Supplementary file 19 — Expanded View Figures [file 44319_2026_720_MOESM19_ESM.pdf]

Expanded View Figures

Figure EV1. Confirmation of Alexa Fluor 555 labeling of iC3b.

(**A**) Ponceau Stain of 8 µg of AF-iC3b, either before spinning down or after a 60 s spin down. (**B**) Example images of cells plated on FN or AF-iC3b treated with FBS (vehicle control), IgG (negative control), αM, or β2 pre- and post-wash for adhesion assay. Scale bar represents 250 µm. (**C**) Percent of cells lost in the process of washing wells in the adhesion assay. (**D**) Percent of cells from pre-washing remaining in post-wash images. (**E**) The total number of alive cells in the pre-wash images. (**F**) Normalized mean difference in intensity of iC3b clearance from the bottom of 10 µg/mL iC3b-coated dishes by BV-2 cells treated with FBS, IgG, αM, or β2 antibodies. Normalization was done to the FBS condition. (**G**) Percent phagocytosis of iC3b-opsonized beads in BV-2 cells at 2 h, after treatment with CR3 blocking antibodies or normal IgG. $N = 3$ experiments (biological replicates) for the antibody adhesion assay (**C–E**), $N = 2$ experiments (biological replicates) for the iC3b uptake with antibody treatment (**F**), and $N = 3$ experiments (biological replicates) for the phagocytosis with antibody assay (**G**). For (**C–E**), statistical analysis was assessed using two-way ANOVA tests. For (**G**), statistical analysis was assessed using ordinary one-way ANOVA. Error bars represent the mean and SEM in all graphs. Values for *p* are as follows: (**C**) ns = nonsignificant, $*p = 0.0108$. (**D**) ns nonsignificant, $**p = 0.0097$. (**E**) ns nonsignificant. (**G**) ns nonsignificant, $****p < 0.0001$, $*p = 0.0396$.

This is a U.S. Government work and not under copyright protection in the US; foreign copyright protection may apply

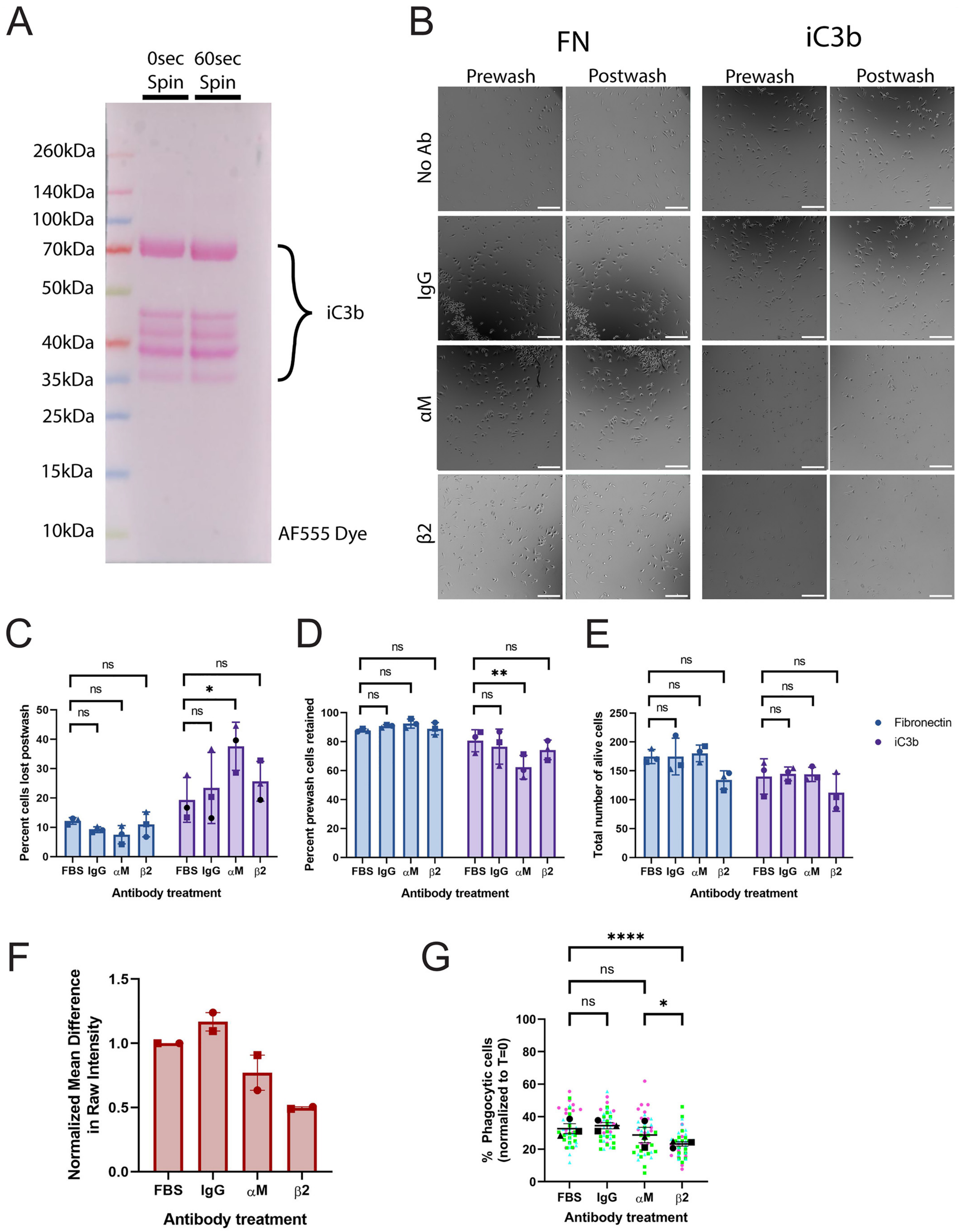
A
0sec Spin
60sec Spin
260kDa
140kDa
100kDa
70kDa
50kDa
40kDa
35kDa
25kDa
15kDa
10kDa
iC3b
AF555 Dye
B
FN
iC3b
Prewash
Postwash
No Ab
IgG
αM
β2
C
Percent cells lost postwash
Antibody treatment
D
Percent prewash cells retained
Antibody treatment
E
Total number of alive cells
Antibody treatment
Fibronectin
iC3b
F
Normalized Mean Difference in Raw Intensity
Antibody treatment
G
% Phagocytic cells (normalized to T=0)
Antibody treatment
FBS
IgG
αM
β2

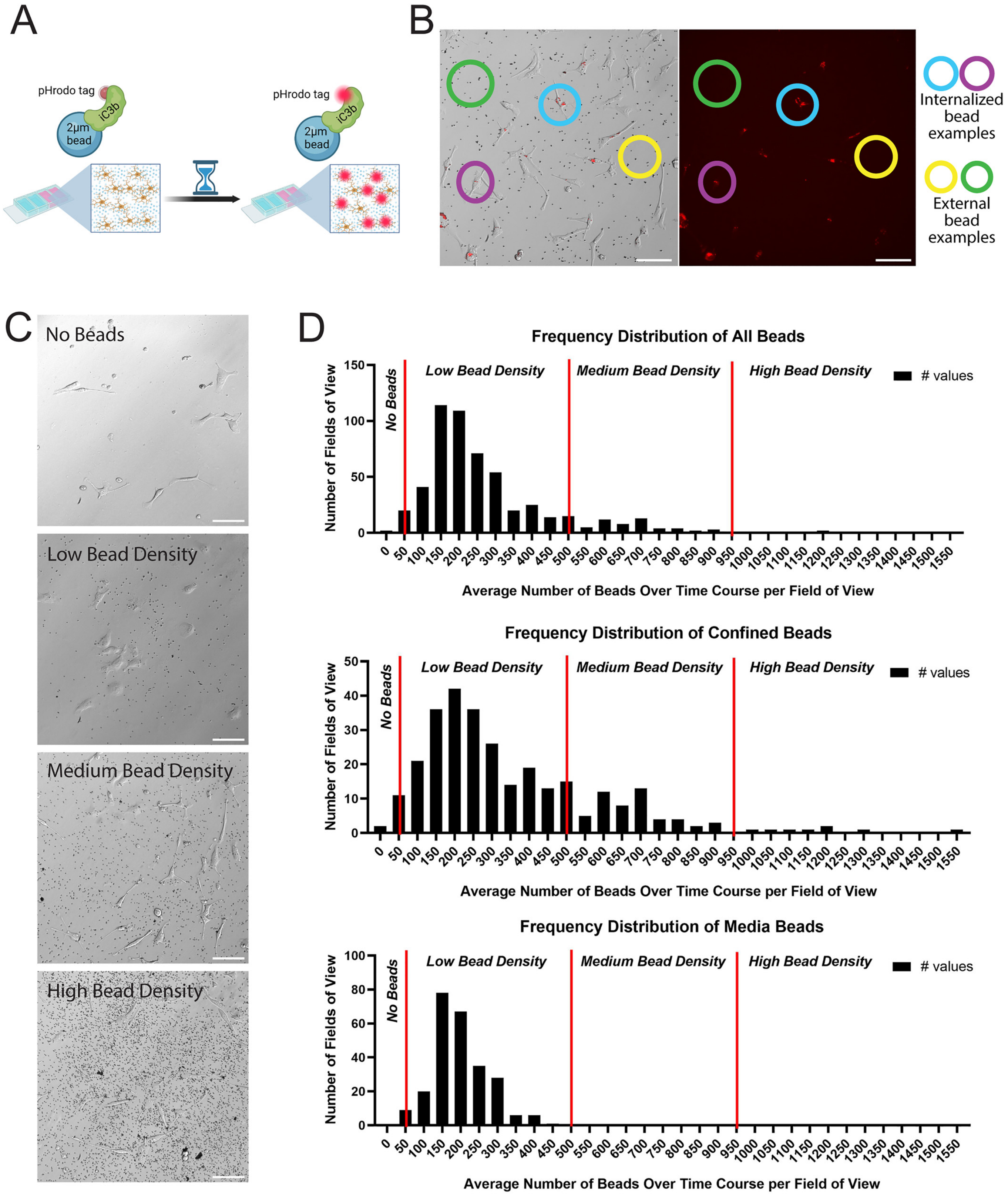

 This is a U.S. Government work and not under copyright protection in the US; foreign copyright protection may apply

◀ **Figure EV2. Strategy to control for bead density.**

(**A**) Schematic depicting phagocytic bead labeling and the pHrodo tag fluorescing inside cells but not externally. (**B**) Duplication of Fig. 1J. Examples of either internalized (blue and purple circles) or external (yellow and green circles) pHrodo-red staining outlined in both composite and pHrodo only images. Scale bar represents 100 μm. (**C**) Example phase contrast images displaying different bead densities. No beads (top) through high bead density (bottom). Scale bar represents 100 μm. (**D**) Histograms detailing the breakdown of average bead densities per field of view across all experimental runs. Low bead density was classified as any field of view with a bead average between 50 and 500 beads; medium bead density was 500 to 950 beads; high bead density was any field of view average above 950 beads. Low bead densities in confined images most closely matched the density of typical media images.

This is a U.S. Government work and not under copyright protection in the US; foreign copyright protection may apply

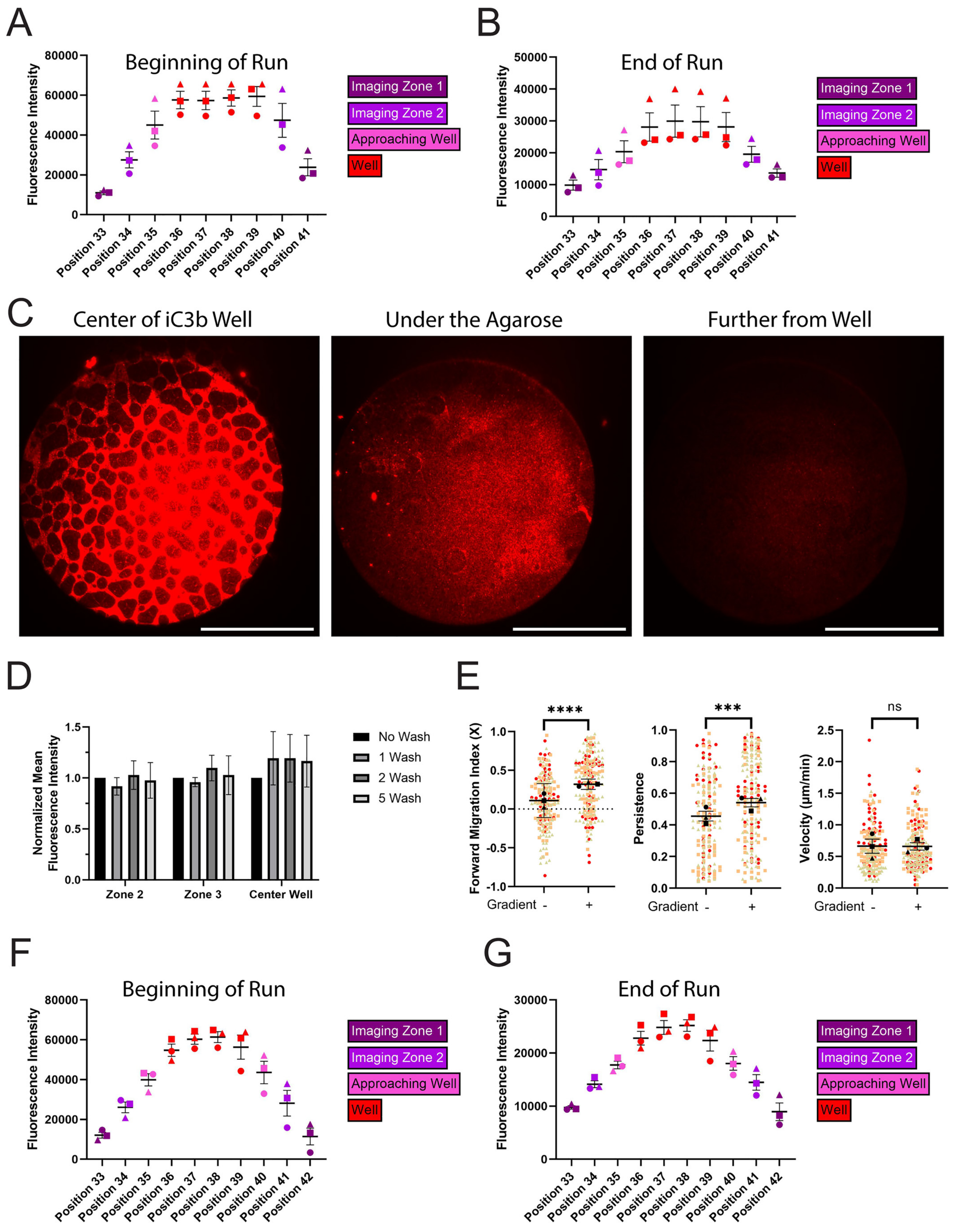

 This is a U.S. Government work and not under copyright protection in the US; foreign copyright protection may apply

Figure EV3. Examining consistency of AF-iC3b labeling during haptotactic assays.

(**A**, **B**) Measurement of the mean fluorescent intensity during haptotaxis runs of the AF-iC3b label spanning from the Zone 1 of one cell well across the center well to the Zone 1 section of the second well. Measurements are color-coded by position (see legend), and symbols represent the three haptotaxis runs. (**A**) corresponds to the beginning of the runs, and (**B**) corresponds to the 24-hour point of the run. (**C**) TIRF images of the glass bottom of the haptotaxis apparatus to demonstrate the binding of AF-iC3b to the glass and the gradient as the signal diminishes, the further from the center well the image is taken. Scale bars represent 50 μm. (**D**) Normalized iC3b mean fluorescence intensity for dishes that received 0 washes, 1 wash, 2 washes, or 5 washes with PBS after 1 h of iC3b coating. Positions imaged are relative to the imaged zones from the cell well. (**E**) Measurements for FMIx (left), persistence (center), and velocity (right) for cells migrating during 125 μM CK-689 treatment. (**F**, **G**) These graphs are the same as (**A**, **B**), but with the CK-666 haptotaxis runs. $N = 3$ experiments (biological replicates) for all graphs; $n = 60$ cells per experiment for (**E**). Values for (**A**, **B**, **D**, **F**, **G**) were taken at 1 image per position. Statistical analysis was assessed using the Mann–Whitney test. Error bars represent means and SEM in all graphs, except for (**E**) left, which is the mean and 95% CI (**E** center and E right are SEM). Values for p are as follows: **E** left) ****$p < 0.0001$. **E** center) ***$p = 0.0004$. **E** right) ns nonsignificant.

This is a U.S. Government work and not under copyright protection in the US; foreign copyright protection may apply

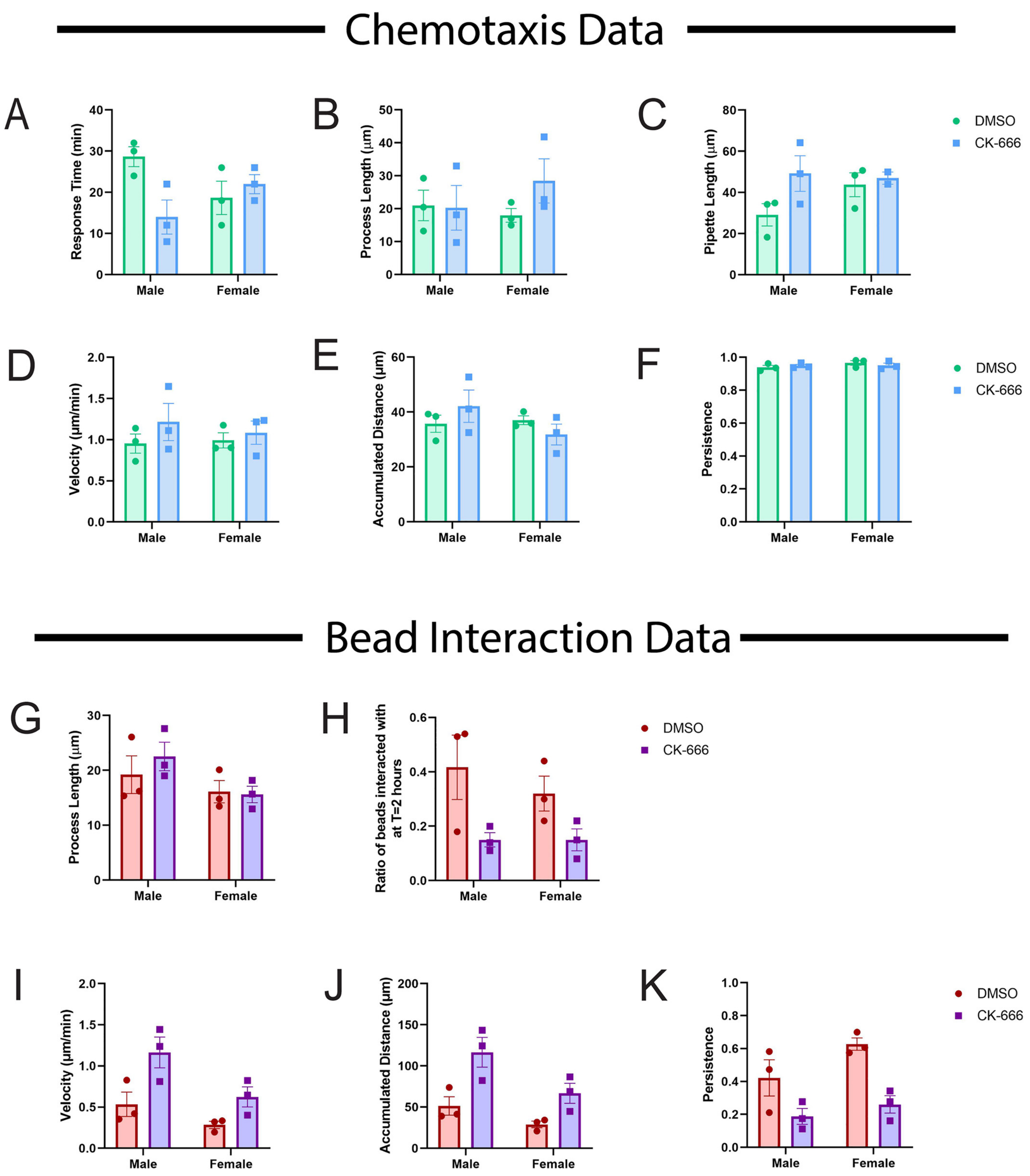

Figure EV4. Lack of microglial sex differences in situ in response to Arp2/2 complex inhibition.

(A–C) ATP results of (Fig. 5B-D) broken down into male and female specific results to examine sex differences. **(D–F)** ATP results of (Fig. 5E–G) broken down into male and female specific results to examine sex differences. **(G, H)** Results from (Fig. 6C, D) were broken down into male and female mice to examine sex differences. **(I–K)** Results of (Fig. 6E–G) are broken down into male and female specific results to examine sex differences. $N = 6$ experiments for each graph (3 male; 3 female biological replicates). Colors for vehicle or CK-666 treatment are clearly marked. Error bars represent SEM in all graphs.

This is a U.S. Government work and not under copyright protection in the US; foreign copyright protection may apply
